# Robinson’s Fibrous and Textile Metallic Filling

**Published:** 1883-07

**Authors:** 


ROBINSON'S FIBROUS AND TEXTILE METALLIC FILLING.

Nearly a year ago some of this material was placed in our hands for trial, since which time we have been employing it with constantly increasing satisfaction, until it has entirely superseded the use of tin foil in our practice. Tin as a material for filling teeth has been too much neglected of late. This material not only possesses all the advantages of that excellent metal, but it has good points that are peculiarly its own. It condenses easily, and gold readily unites with it, so that the advocates of a union of metals in the same filling have here a preparation especially adapted to their wants. For filling large approximal cavities extending beneath the gum, in which the cervical border is uneven or fractured, or those in which it is extremely difficult to prevent the intrusion of fluids into the cavity, a piece of the fibrous filling makes an excellent base upon which to build the gold, for it not only condenses into the inequalities readily, but it enables one to use the slight antiseptic qualities of the material. For the lining of rubber dentures we have not yet had occasion to use it.

